# Moss‐made pharmaceuticals: from bench to bedside

**DOI:** 10.1111/pbi.12401

**Published:** 2015-05-25

**Authors:** Ralf Reski, Juliana Parsons, Eva L. Decker

**Affiliations:** ^1^ Plant BiotechnologyFaculty of BiologyUniversity of FreiburgFreiburgGermany; ^2^ FRIAS ‐ Freiburg Institute for Advanced StudiesFreiburgGermany; ^3^ BIOSS ‐ Centre for Biological Signalling StudiesFreiburgGermany

**Keywords:** ADCC, biobetter, knockout moss, Morbus Fabry, moss bioreactor, *Physcomitrella patens*

## Abstract

Over the past two decades, the moss *Physcomitrella patens* has been developed from scratch to a model species in basic research and in biotechnology. A fully sequenced genome, outstanding possibilities for precise genome‐engineering via homologous recombination (knockout moss), a certified GMP production in moss bioreactors, successful upscaling to 500 L wave reactors, excellent homogeneity of protein glycosylation, remarkable batch‐to‐batch stability and a safe cryopreservation for master cell banking are some of the key features of the moss system. Several human proteins are being produced in this system as potential biopharmaceuticals. Among the products are tumour‐directed monoclonal antibodies with enhanced antibody‐dependent cytotoxicity (ADCC), vascular endothelial growth factor (VEGF), complement factor H (FH), keratinocyte growth factor (FGF7/KGF), epidermal growth factor (EGF), hepatocyte growth factor (HGF), asialo‐erythropoietin (asialo‐EPO, AEPO), alpha‐galactosidase (aGal) and beta‐glucocerebrosidase (GBA). Further, an Env‐derived multi‐epitope HIV protein as a candidate vaccine was produced, and first steps for a metabolic engineering of *P. patens* have been made. Some of the recombinant biopharmaceuticals from moss bioreactors are not only similar to those produced in mammalian systems such as CHO cells, but are of superior quality (biobetters). The first moss‐made pharmaceutical, aGal to treat Morbus Fabry, is in clinical trials.

## Introduction

Most recombinant biopharmaceuticals are complex human glycoproteins. Therefore, the benchmarks for any production system are mammalian cell factories, especially CHO cells, which were derived from Chinese hamster ovaries (Beck *et al*., 2008; Durocher and Butler, 2009). The use of plants as alternative expression hosts is on the rise because their cultivation may be easier, less expensive and *per se* excludes possible contaminations of the product with infectious agents deleterious to the patient, which should make downstream processing and safety tests more straightforward and thus less expensive (Fischer *et al*., 2004; other articles in this special issue). Consequently, several plant‐made pharmaceuticals (PMPs) are already in clinical studies (Paul and Ma, 2011; Sabalza *et al*., 2014), and the first product, Taliglucerase alfa, a beta‐glucocerebrosidase to treat Morbus Gaucher (Shaaltiel *et al*., 2007), was released to the market in 2012 by Pfizer/Protalix.

In comparison with the benchmarks, three major challenges remain before plants can be widely used as alternative cell factories:

1. The amounts of recombinant products need to be enhanced by several techniques.
2. Although the core di‐antennary N‐glycan‐structure on glycoproteins is the same between human and plants, specific differences do exist and can affect stability, efficacy and tolerance by the patient's immune system.
3. The prerequisite for approval is a production of the drug according to good manufacturing practice (GMP) guidelines, which requires production in self‐contained systems such as bioreactors.

Within this special issue on Molecular Farming, we concentrate on the production of PMPs utilizing the moss *Physcomitrella patens* as a production host. Broader information on specific aspects of this topic can be found in previous reviews. The basic concept was described in Decker and Reski (2004), different aspects of glycoprotein production were discussed in Decker and Reski (2007), and the production process is reviewed in Decker and Reski (2008). Detailed reviews on glyco‐engineering aspects can be found in Decker and Reski (2012) and in Decker *et al*. (2014). Instead, we aim here at providing a concise review of the system, the established enabling technologies, and the most current update on the status of produced potential biopharmaceuticals.

## The moss system

Bryophyte (comprising mosses, hornworts and liverworts) research has a long history with several important contributions to basic science (reviewed in Reski, 1998a). From an evolutionary point of view, mosses are situated halfway between single‐celled algae and complex seed plants, which are divergent by an evolutionary distance of about one billion years (Lang *et al*., 2008). As in algae, the dominant phase of moss development is haploid (gametophytic), while the dominant phase in all seed plants is at least diploid (sporophytic). Mosses can be grown in self‐contained systems like Petri dishes, Erlenmeyer flasks or bioreactors in pure mineral media without any organic additions such as antibiotics, carbon sources or growth regulators and under highly controlled environmental conditions (Cerff and Posten, 2012; Hohe and Reski, 2005; Hohe *et al*., 2002a). Even under these conditions, the moss *P. patens* can complete its life cycle with the release of persistent spores. Sexual reproduction, however, is only initiated under low temperature and short day conditions (Hohe *et al*., 2002b). Therefore, no spores are formed under production conditions.

## Enabling technologies

Vector‐free genetic transformation of *P. patens* has been established by conferring antibiotic resistance to wild‐type moss (Schaefer *et al*., 1991). Shortly afterwards, it became evident that this moss is suitable for precise gene targeting (GT) via homologous recombination (HR) (Kammerer and Cove, 1996; Schaefer and Zrÿd, 1997). Because of its outstanding high rate of HR and the haploid nature of its dominant growth phase, GT was subsequently used to disrupt genes of interest and infer gene functions from these knockout mosses in a reverse genetics approach without complex backcrosses (Girke *et al*., 1998; Strepp *et al*., 1998) and even allowing high‐throughput analyses (Egener *et al*., 2002). Protocols for culture media, protoplast isolation and regeneration, and for PEG‐mediated transformation have been optimized, allowing for the simultaneous disruption of multiple different genes in one experiment (Hohe and Reski, 2002; Hohe *et al*., 2004; Schween *et al*., 2003, 2005). The high rate of GT is a clear advantage for glyco‐engineering of moss when compared to similar approaches in seed plants (Reski, 1998b; Schaefer and Zrÿd, 2001). However, as alternatives to the complete destruction of genes via GT (Kamisugi *et al*., 2006), methods for base‐specific genome alterations and for creating fusion proteins in the original genomic context (Mosquna *et al*., 2009; Mueller *et al*., 2014) as well as for a gradual down‐regulation of gene expression via artificial microRNAs (Khraiwesh *et al*., 2008, 2010) have also been established in *P. patens*.

A wide variety of promoter elements have been evaluated for transgene expression in moss, including bacterial (Reutter *et al*., 1998), endogenous (Jost *et al*., 2005) and seed plant promoters (Holtorf *et al*., 2002). Collectively, the set of suitable promoter fragments allows for a control of gene expression over three orders of magnitude (Horstmann *et al*., 2004). This includes promoter elements which can be induced by temperature (Saidi *et al*., 2005), chemicals (Kubo *et al*., 2013) or red light (Müller *et al*., 2014). Astonishingly, *P. patens* accepts a wide variety of components of the transcription, translation and secretion machineries, originally developed and optimized for recombinant production in CHO cells (Gitzinger *et al*., 2009). In addition, endogenous signal sequences are used to direct recombinant proteins through the ER, and finally secrete them to the culture medium (Schaaf *et al*., 2005). Alternatively, the product can be integrated into the membrane, thus functionalizing the moss with extracellular catalytic and/or binding activities (Morath *et al*., 2014). Product stability in the medium can be enhanced by stabilizing additives or by the co‐expression of human serum albumin (Baur *et al*., 2005a). A transient expression assay was developed for fast proof‐of‐concept studies (Baur *et al*., 2005b).

The *P. patens* genome comprises 500 Mbp distributed on 27 chromosomes (Reski *et al*., 1994). It has been fully sequenced (Rensing *et al*., 2008) as third plant genome subsequent to the genomes of *Arabidopsis thaliana* and poplar. The full genome information is freely available via www.cosmoss.org and is constantly improved (Zimmer *et al*., 2013). A variety of whole genome transcription profiling data sets were published (e.g. Beike *et al*., 2015; Richardt *et al*., 2010), which have been integrated into a transcriptomic platform allowing analysis of transcript abundances, and thus indirectly promoter activity, under different culture conditions (Hiss *et al*., 2014).

**Figure 1:**
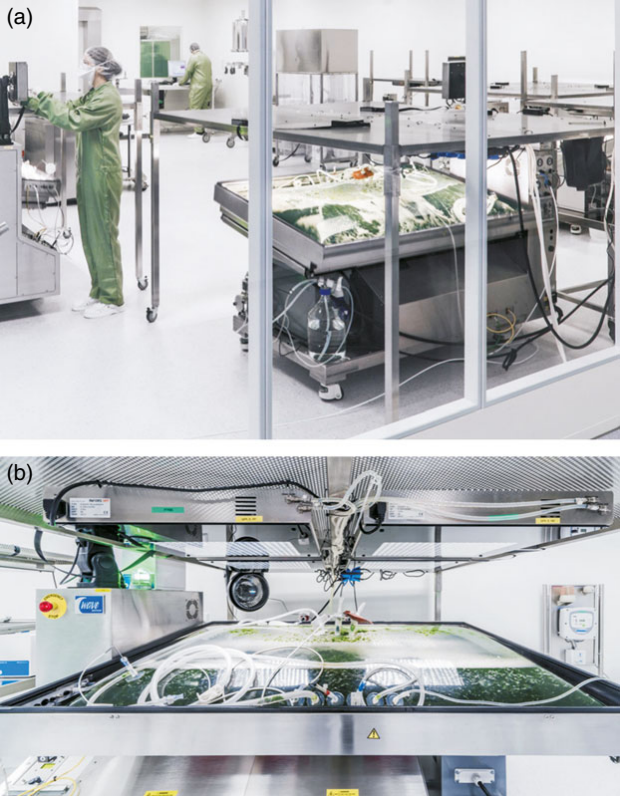
Industrial‐scale, GMP‐compliant disposable wave reactors for the production of biopharmaceuticals expressed in the moss *Physcomitrella patens*. (a) A room equipped with several wave bioreactors. (b) A detailed view on a wave bioreactor and its illumination system. Images are courtesy of Greenovation Biotech GmbH.

For the production of biopharmaceuticals, large‐scale processes have to be implemented. Bioreactors have been used for plant cell cultures (Kieran *et al*., 1997) and transgenic hairy root cultures (Giri and Narasu, 2000) as they provide containment and are easy to control, thus facilitating production under GMP conditions. Different kinds and sizes of photobioreactors have been established for the controlled large‐scale culture of *P. patens*: the first production was achieved in a 2‐L foil‐reactor (Reutter and Reski, 1996), subsequently followed by 5 L,10 L and 20 L stirred glass tanks (Hohe *et al*., 2002a), which are still the ‘working horses’ in the laboratory. A further upscale to 100 L was achieved by the development of tubular photobioreactors (Perner‐Nochta *et al*., 2007). Currently, for commercial production under GMP‐certified conditions, 100 L and 500 L disposable wave reactors are in use (Niederkrüger *et al*., 2014; Figure 1). While plant cell cultures show a high degree of genetic instability, the so‐called somaclonal variation (Larkin and Scowcroft, 1981; Phillips *et al*., 1994), moss tissue culture is genetically stable over long periods of time (von Stackelberg *et al*., 2006), because it relies on differentiated moss plants instead of undifferentiated plant cell cultures.

An important issue in GMP regulations is the molecular characterization of the producing cell factories. Once characterized and approved, subsequent production has to rely on identical clones that have to be stored in master cell banks (MCBs). This can easily be achieved for clonal moss tissues, as they can be stored for years over liquid nitrogen and survive this cryopreservation to 100% even after years (Schulte and Reski, 2004). Probably, such a highly controlled cryopreservation period is only limited by human civilization, because recently a moss has been regrown in the laboratory after surviving 1500 years in the Antarctic permafrost (Roads *et al*., 2014). To facilitate international moss research, noncommercial moss strains are cryopreserved and distributed by the International Moss Stock Center (IMSC) in Freiburg, Germany (www.moss-stock-center.org).

## Precision glyco‐engineering

Post‐translational addition of sugar moieties to proteins occurs in the ER and the Golgi apparatus in a multistep process. These sugars can be attached either to the amide group of asparagine (N‐glycosylation), or the hydroxyl group of serine, threonine, hydroxylysine or hydroxyproline (O‐glycosylation). Whereas consensus sequences for O‐glycosylation are almost unpredictable for mammalian proteins (Julenius *et al*., 2005), they are well defined for N‐glycosylation. In general, the basic structure of N‐glycosylation is conserved between humans and plants. The latter, however, show a higher uniformity of N‐glycosylation patterns between different tissues and across species (Bosch *et al*., 2013). This feature is an additional argument for plants as production hosts, because it implies a higher batch‐to‐batch reproducibility and higher homogeneity of PMP compared to conventional products. Although evolutionary apart from seed plants by 500 million years, the moss *P. patens* performs N‐glycosylation similar to them (Koprivova *et al*., 2003; Viëtor *et al*., 2003). Genes encoding plant‐typical glycosyltransferases were identified and deleted from the moss genome (Koprivova *et al*., 2004; Parsons *et al*., 2012). Subsequently, a mutant was engineered to further ‘humanize’ the moss glycosylation pattern by the expression of a human beta‐1,4‐galactosyltransferase gene. This gene was integrated into the *P. patens* genome by ‘knockin’ into the xylosyltransferase or fucosyltransferase locus, respectively (Huether *et al*., 2005). To avoid unwanted O‐glycosylation of human proteins produced in moss, a gene responsible for prolyl‐hydroxylation was identified and deleted from the genome (Parsons *et al*., 2013). Under laboratory conditions, all these glyco‐mutants do not differ from the wild‐type strain regarding growth and development or productivity and secretion capability.

## Recombinant products

Several proteins have been produced in moss. The as‐yet disclosed products are discussed in the following and are compiled in Table 1. The field started with the expression of the bacterial beta‐glucuronidase (GUS) as a quantifiable reporter protein in 2 L moss bioreactors (Reutter and Reski, 1996). Further reporter proteins that were used to monitor the production process were a bacterial alpha‐amylase (AMY) and the human placental secreted alkaline phosphatase (SEAP) (Gitzinger *et al*., 2009) as well as the F‐actin marker GFP‐talin (Saidi *et al*., 2005). As a product as well as a stabilizing agent for secreted biopharmaceuticals the human serum albumin (HSA) has been co‐expressed in the production process (Baur *et al*., 2005a).

**Table 1:** Compilation of recombinant proteins produced in the moss *Physcomitrella patens*

Abbreviation	Full name	Original expressing organism	Function	MW (kDa)	References
GUS	Beta‐glucuronidase	*Escherichia coli*	Hydrolyses β‐glucuronic acid residues from glucuronides giving rise to coloured products; reporter protein	68	Reutter and Reski, 1996
VEGF	Vascular endothelial growth factor	Human	Plays an important role in angiogenesis by inducing the proliferation of endothelial cells	28	Gorr *et al*., 2001; Koprivova *et al*., 2004; Baur *et al*., 2005a
HSA	Human serum albumin	Human	Used as stabilizing agent for therapeutic proteins	66	Baur *et al*., 2005b
GFP‐talin	Green fluorescent protein‐talin	*Aequorea victoria*/mouse	Used to label F‐actin filaments; reporter protein	48	Saidi *et al*., 2005
IgG1 IGN314	Glyco‐optimized version of antibody IGN311	Human	Antibody recognizing tumour‐associated glycosylation pattern Lewis Y	150	Schuster *et al*., 2007; Kircheis *et al*., 2012
AEPO	Asialo‐erythropoietin	Human	Displays anti‐apoptotic activity. Potential treatment of stroke	30	Weise *et al*., 2007; Parsons *et al*. 2012
SEAP	Placental secreted alkaline phosphatase	Human	Dephosphorylation; reporter protein	75	Gitzinger *et al*., 2009
AMY	Alpha‐amylase	*Bacillus stearother‐mophilus*	Hydrolyses internal alpha‐(1,4)‐D‐glucosidic linkages on starch, glycogen and related polysaccharides.	64	Anterola *et al*., 2009
Taxadiene synthase	Taxadiene synthase	*Taxus brevifolia*	Enzyme responsible for the synthesis of a precursor of paclitaxel, a widely used anticancer drug.	75	Gitzinger *et al*., 2009
FH	Complement factor H	Human brevifolia	Key regulator of the alternative pathway of complement activation. Potential treatment of atypical haemolytic uremic syndrome or C3 glomerulopathies	155	Büttner‐Mainik *et al*., 2011
AGal	Alpha‐galactosidase	Human	Catalyses the cleavage of terminal galactose from ceramide trihexosides. Enzyme replacement therapy in Fabry disease (lysosomal storage disease)	46	Niederkrüger *et al*., 2014
GBA	Beta‐glucocerebrosidase	Human	Catalyses the cleavage of glucose from glucocerebrosides. Enzyme replacement therapy in Gaucher disease (lysosomal storage disease)	60	Niederkrüger *et al*., 2014
FGF7/KGF	Keratinocyte growth factor	Human	Promotes proliferation of keratinocytes. Re‐epithelialization of wounds. Used in many *in vitro* mammal cell cultures	19–28	Niederkrüger *et al*., 2014
EGF	Epidermal growth factor	Human	Promotes proliferation of epithelial cells	6	Niederkrüger *et al*., 2014
HGF	Hepatocyte growth factor	Human	Potent mitogen. It regulates cell proliferation and morphogenesis. It plays a key role in tissue regeneration. Used in *in vitro* mammal cell culture	100	Niederkrüger *et al*., 2014
PTS	Patchoulol synthase	*Pogostemon cablin*	Synthesis of patchoulol, a sesquiterpenoid used as fragrance	64	Zhan *et al*., 2014
STS	Alpha/beta‐santalene synthase	*Santalum album*	Synthesis of alpha/beta‐santalol, a sesquiterpenoid used as fragrance	66	Zhan *et al*., 2014
Poly HIV	Multi‐epitope fusion protein from the human immunodeficiency virus	Artificial/HIV	HIV vaccine candidate	33	Orellana‐Escobedo *et al*., 2015

Mosses contain far more genes involved in secondary metabolism than seed plants (Rensing *et al*., 2007). Some of these metabolites possess well‐known human health benefits (Beike *et al*., 2014; Reski and Frank, 2005). Therefore, one side‐aspect of the field is the metabolic engineering of moss to enhance the production of secondary metabolites with commercial value. A breakthrough in this respect was the expression of a taxadiene synthase from *Taxus brevifolia* (Anterola *et al*., 2009), an enzyme responsible for the synthesis of a precursor of paclitaxel, a widely used anticancer drug (Baird *et al*., 2010). Another target for engineered mosses is the fragrance industry. In this respect, a patchoulol synthase and an alpha/beta‐santalene synthase have been expressed in *P. patens* (Zhan *et al*., 2014). Patchoulol and alpha/beta‐santalol are two sesquiterpenoids used in fragrances (Faraldos *et al*., 2010; Jones *et al*., 2011).

The first human protein produced in the moss system was the vascular endothelial growth factor (VEGF) (Baur *et al*., 2005a; Gorr *et al*., 2001; Koprivova *et al*., 2004), which has a central function in angiogenesis and in cancer (Goel and Mercurio, 2013; Roskoski, 2007). Human complement factor H (FH) is the key regulator of the alternative pathway of complement activation and a protectant against oxidative stress (Weismann *et al*., 2011). Because it is a large protein of 155 kDa and contains 40 disulphide bonds, it is a difficult‐to‐express protein. Therefore, attempts are ongoing to produce bioactive but truncated versions (mini FH) in insect cells (Hebecker *et al*., 2013). Full‐length FH has been successfully produced in moss with biological activity *in vitro* (Büttner‐Mainik *et al*., 2011). After having confirmed full biological activity in different bioassays, this protein will be further evaluated in FH‐deficient knockout mice. FH supply is a potential treatment for kidney diseases such as atypical haemolytic uremic syndrome (aHUS) and C3 glomerulopathies (Sethi *et al*., 2012) and for age‐related macular degeneration (AMD) (Bradley *et al*., 2011). A moss‐made FH may be a cost‐effective and more compliant alternative to the monoclonal antibody eculizumab, which is limited to the treatment of aHUS, has severe side effects (Schmidtko *et al*., 2013) and, moreover, is the most expensive biopharmaceutical worldwide with treatment costs of about 400,000 Euro per year and patient.

Several human growth factors (FGF7/KGF, EGF and HGF) that are used in mammalian cell culture have been produced in the moss system (Niederkrüger *et al*., 2014). FGF7/KGF (keratinocyte growth factor) is the first commercially available moss‐made human protein, intended for *in vitro* use (www.greenovation.com). Based on these experiences, moss has been suggested as a potential production host for vaccines (Rosales‐Mendoza *et al*., 2014). As no adverse effects of moss consumption are known, vaccine‐producing moss may be directly administered as an oral vaccine. Thus, expensive protein purification could be avoided. The first moss‐made candidate vaccine is a chimeric Env‐derived HIV multi‐epitope protein that is immunogenic in mice (Orellana‐Escobedo *et al*., 2015).

## Biobetters from moss

Plants are prevalently discussed as alternative production hosts because of lower costs and increased safety. It is believed that they may produce human proteins in a similar way as, for example, CHO cells do. For the production of such biosimilars, extensive glyco‐engineering approaches have to be made as discussed above and in more detail in Decker *et al*. (2014). However, examples are emerging that these inherent differences between plants and mammals may favour plant cell factories, as they, at least in some cases, produce superior biopharmaceuticals, so‐called biobetters. A discussion on biosimilars and biobetters can be found in Beck (2011). A glyco‐optimized monoclonal antibody (IgG1 IGN314) that was developed to recognize tumour‐associated glycosylation patterns (Lewis Y) was produced in moss. It was 40 times more effective at inducing lysis in three different tumour cell lines than the same antibody produced in CHO cells (Schuster *et al*., 2007). Unlike mammals, plants lack the alpha‐1,6‐linked fucose residue at the base of the bi‐antennary N‐glycan structure. The moss‐made antibody lacking this sugar moiety was obviously far more efficient in antibody‐dependent cellular cytotoxicity (ADCC) than the CHO‐derived antibody (Kircheis *et al*., 2012) and thus a clear biobetter.

Erythropoietin (EPO) is the major hematopoietic hormone (cytokine) and has multiple effects besides the well‐known induction of red blood cell maturation in bone marrow. Additional effects in kidney function, angiogenesis, neurogenesis, the immune response and in preventing apoptosis are well documented (Lombardero *et al*., 2011). Besides that, EPO has an inglorious use in illegal doping activities. Functional EPO has been produced in moss (Weise *et al*., 2007). The protein was, however, decorated with so‐called Lewis A (Le^a^) structures, as was the similar product from *Nicotiana benthamiana* (Castilho *et al*., 2013). Such Le^a^ epitopes are biomarkers for certain types of cancer (Rho *et al*., 2014) and, thus, should be avoided on PMPs. A single gene responsible for the synthesis of Le^a^ epitopes in *P. patens* was identified and deleted from the moss genome. The resulting asialo‐EPO (AEPO) was of a remarkably high uniformity with almost only one glycosylation form and devoid of Le^a^ epitopes and any other plant‐typical glyco‐epitopes (Parsons *et al*., 2012). Such an asialo‐EPO does not promote the maturation of red blood cells, and thus cannot be abused for doping, but exerts neuroprotective and anti‐apoptotic functions, and therefore could be beneficial in stroke treatment without the potential thromboembolic risk of EPO (Kaneko *et al*., 2013; Sirén *et al*., 2009). To enhance the safety and efficiency of moss‐made asialo‐EPO even further, a gene was identified and eliminated from the moss genome that is responsible for an undesired non‐human prolyl‐hydroxylation. In plants, this hydroxyproline is the anchor site for plant‐typical O‐glycosylation, which is also undesired in PMPs (Parsons *et al*., 2013). Thus, moss‐made asialo‐EPO appears to be a safe biobetter for a variety of indications.

Morbus Gaucher and Morbus Fabry are two orphan lysosomal storage diseases with severe implications (Boustany, 2013; Lieberman *et al*., 2012), which can be treated by an enzyme replacement therapy (Beck, 2010). Both enzymes, human alpha‐galactosidase (aGal) for Fabry and beta‐glucocerebrosidase for Gaucher disease, are being produced in moss. A detailed analysis of glycan structures from different batches proved a higher homogeneity and a significantly enhanced batch‐to‐batch stability compared to commercially available drugs that are produced in mammalian cell lines (Niederkrüger *et al*., 2014). Thus, the production system itself is able to produce superior biopharmaceuticals. In addition, moss‐made aGal lacks the terminal mannose phosphorylation and thus is imported into cells via mannose receptors and not mannose‐6 phosphate receptors, yielding better pharmacokinetics in Fabry mice. Moss‐made aGal has successfully passed toxicity testing and is currently in clinical trials (www.greenovation.com).

## Conclusions

A wide variety of human glyco‐proteins are currently produced in mammalian cell factories such as CHO cells. With the advent of personalized medicine, the demand for such recombinant biopharmaceuticals will increase steeply. Plant‐based systems are being developed as cost‐effective and safe alternative production hosts. Among those, the moss system has unique advantages because it combines the best of both worlds. Some moss‐made pharmaceuticals have superior quality compared to conventional products from insect or mammalian cell factories, as evidenced by a forty times better ADCC and better batch‐to‐batch reproducibility with regard to protein glycosylation. Preclinical and clinical trials are underway to evaluate whether the moss system is suitable to provide next‐generation biopharmaceuticals as clear biobetters.

## Conflict of interest

R.R. is an inventor of the moss bioreactor and a founder of Greenovation Biotech GmbH. He currently serves as advisory board member of this company. E.L.D., J.P. and R.R. are inventors of patents and patent applications related to the topics discussed here. The Chair of Plant Biotechnology at the University of Freiburg, headed by R.R., developed and hosts the resources www.cosmoss.org and www.moss-stock-center.org.
